# THEORIES OF CREATIVITY AND CRAFTS FOR OLDER PEOPLE

**DOI:** 10.1093/geroni/igad104.0140

**Published:** 2023-12-21

**Authors:** Carolyn Adams-Price

**Affiliations:** Mississippi State University, Mississippi State, Mississippi, United States

## Abstract

Although gerontologists have increased their discussions of the benefits of creative activities for older adults, some aspects of late life creativity have been discussed very little. Two aspects of late life creativity that have received little attention include 1) long-term participation in a particular creative domain, resulting in learning and the development of skills, and 2) the participation in culturally-meaningful crafts, especially those that connect people or result in a cultural legacy. Referring to examples from diverse cultures, we will discuss how theories of adult development and theories of creativity can be merged into a framework for studying the significance of participation in culturally-significant crafts for diverse older adults. Erikson’s theory of adult development and Glaveanu’s sociocultural theory of creativity will be used as a framework to explore the meaning and potential of participation in culturally-significant crafts for positive development in late life. We will discuss how practice leads to learning and mastery, how identity benefits from creating culturally-significant crafts, how creating in groups (or with an audience) leads to greater engagement, and how the creation of sharable products can lead to a meaningful legacy for some older creators. Examples from diverse cultures will be included, including from the two previous papers, and from older rural African American women who quilt.

